# Independent and joint associations between the triglyceride-glucose index and NT-proBNP with the risk of adverse cardiovascular events in patients with diabetes and acute coronary syndrome: a prospective cohort study

**DOI:** 10.1186/s12933-023-01890-9

**Published:** 2023-06-26

**Authors:** Man Wang, Li Zhou, Wen Su, Wenxi Dang, Hongwei Li, Hui Chen

**Affiliations:** 1 Department of Cardiology, Cardiovascular Center, Beijing Friendship Hospital, Capital Medical University, No.95, Yongan Road, Xicheng District, Beijing, 100050 People’s Republic of China; 2 Beijing Key Laboratory of Metabolic Disorder Related Cardiovascular Disease, Beijing, China

**Keywords:** Triglyceride-glucose index, NT-proBNP, Acute coronary syndrome, Diabetes, Joint association

## Abstract

**Background:**

Elevated triglyceride-glucose (TyG) index and N-terminal pro-B-type natriuretic peptide (NT-proBNP) are independently associated with increased risk of major adverse cardio-cerebral events (MACCEs) in diabetic patients with the acute coronary syndrome (ACS), but have not been evaluated jointly. We sought to investigate the independent and joint association of the TyG index and NT-proBNP with MACCEs risk.

**Methods:**

Data from 5046 patients with diabetes and ACS were recorded in the Cardiovascular Center Beijing Friendship Hospital Database Bank between 2013 and 2021, including measurements of fasting triglycerides, plasma glucose, and NT-proBNP. The TyG index was calculated as Ln (fasting triglycerides [mg/dL] × fasting plasma glucose [mg/dL]/2). Associations of the TyG index and NT-proBNP with MACCEs risk were assessed using flexible parametric survival models.

**Results:**

During 13589.9 person-years of follow-up, 985 incident MACCEs of the 5046 patients (65.6 years of age and 62.0% men) were observed. Elevated TyG index (HR: 1.18; 95% CI 1.05‒1.32 per 1 unit increase) and NT-proBNP categories (HR: 1.95; 95% CI: 1.50‒2.54 for > 729 pg/ml compared to < 129 pg/ml) were independently associated with MACCEs risk in the fully adjusted model. According to the joint categories of the TyG index and NT-proBNP, patients with the TyG index > 9.336 and NT-proBNP > 729 pg/ml were at the highest risk of MACCEs (HR: 2.45; 95% CI 1.64‒3.65) than the ones with TyG index < 8.746 and NT-proBNP < 129 pg/ml. The test for interaction was not significant (*P*_interaction_ = 0.49). Incorporating these two biomarkers into the established clinical model, the Global Registry of Acute Coronary Events (GRACE) risk score, resulted in a significant improvement in risk stratification.

**Conclusions:**

The TyG index and NT-proBNP were independently and jointly associated with the risk of MACCEs in patients with diabetes and ACS, suggesting that patients with both markers elevated should be aware of the higher risk in the future.

**Supplementary Information:**

The online version contains supplementary material available at 10.1186/s12933-023-01890-9.

## Introduction

Acute coronary syndrome (ACS) is a severe manifestation of coronary artery disease (CAD) and is one of the leading causes of morbidity and mortality in China [1]. Diabetes is one of the major risk factors for CAD development [2] and is significantly correlated with multivessel disease [3]. Additionally, diabetes is significantly associated with an increased risk for subsequent adverse cardiovascular events in patients with ACS [4, 5]. Thus, for patients with diabetes and ACS, early and precise risk stratification to identify patients at a high risk of developing future adverse cardiovascular outcomes is crucial.

Insulin resistance (IR) is a key pathophysiology pathway for the development of type 2 diabetes mellitus (T_2_DM) and cardiovascular disease [6–8]. The triglyceride-glucose index (TyG) index is a reliable marker of IR that combines fasting triglycerides (TGs) and fasting plasma glucose (FPG) [9]. The accuracy of the TyG index has been tested in several studies using the hyperinsulinemic-euglycemic clamp (HIEC) and homeostasis model assessment of insulin resistance (HOMA-IR) [10–12]. The TyG index is significantly associated with an increased risk of cardiovascular events in the general population and cardiovascular disease patients according to numerous studies [13–15]. Wang et al. also revealed that the TyG index was an independent predictor of future major adverse cardiovascular events in patients with diabetes and ACS independent of known cardiovascular risk factors [16].

N-terminal pro-B-type natriuretic peptide (NT-proBNP) is a neurohormone released from cardiomyocytes in response to increased ventricular wall stress, hypertrophy, and volume overload [17]. Its prognostic usefulness for adverse cardiovascular outcomes in the spectrum of ACS is well established [18]. In contradiction, a growing body of research has revealed NT-proBNP to have an inverse association with metabolic factors such as IR, body mass index (BMI), and blood lipid levels [19–22]. HOMA-IR, which is mostly used in the assessment of the association of IR and NT-proBNP, was found to be negatively correlated with NT-proBNP [19, 22, 23]. However, no previous study has evaluated the relationship of NT-proBNP with the IR marker of the TyG index. Furthermore, it has been reported that an increased B-type natriuretic peptide level would accelerate lipolysis in adipose tissue, resulting in IR improvements [24]. Therefore, patients with established diabetes and cardiovascular disease may be confused about how the TyG index and NT-proBNP affect the prognosis as a result of this inverse association. No studies have systematically evaluated the independent and joint associations of the TyG index and NT-proBNP with the prognosis of patients with diabetes and ACS.

In this study, we sought to investigate the independent and joint association of the TyG index and NT-proBNP with the risk of adverse cardiovascular events in a large, Chinese cohort of patients with diabetes and ACS.

## Methods

### Study population

**Fig. 1:**
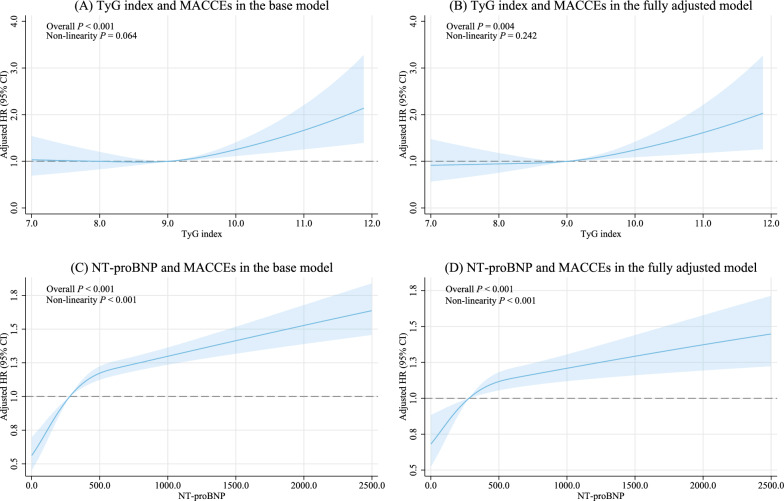
Restricted cubic spline analyses for association of the TyG index and NT-proBNP with the risk of MACCEs in the base and fully adjusted model

This study was a prospective, observational cohort study. A total of 15,330 consecutive patients diagnosed with ACS were admitted to Beijing Friendship Hospital from January 2013 to January 2021, all of whom were documented in the Cardiovascular Center Beijing Friendship Hospital Database Bank (CBDBANK). Exclusion criteria included: (1) absence of NT-proBNP, FPG, or TGs, (2) without diabetes, (3) severe liver dysfunction (alanine ≥ 5 times the upper reference limits), severe renal insufficiency (estimated glomerular filtration rate [eGFR] < 30 ml/min/1.73m^2^), or kidney replacement treatment, (4) severe acute infection or malignancy, and (5) previous coronary artery bypass grafting (CABG), cardiogenic shock or severe heart failure (left ventricular ejection fraction [LVEF] < 30%). ACS was defined as including either unstable angina pectoris (UAP), non-ST-segment elevation myocardial infarction (NSTEMI), or ST-segment elevation myocardial infarction (STEMI). Diabetes was defined as having been previously diagnosed, taking anti-diabetic drugs, FPG ≥ 7.0 mmol/L, or glycated hemoglobin (HbA1c) ≥ 6.5%. Cardiogenic shock was defined as systolic blood pressure (SBP) < 90 mmHg for ≥ 30 min or catecholamines to maintain SBP > 90 mmHg, and clinical pulmonary congestion and impaired end-organ perfusion (altered mental status, cold/clammy skin and extremities, urine output < 30 ml/h, or lactate > 2.0 mmol/L), or a class IV rating based on the Killip classification. A total of 5046 patients with diabetes and ACS were included in this study (Additional file 1**: **Fig. 1).

The study was approved by the Ethics Committee of Beijing Friendship Hospital affiliated to Capital Medical University (2021-P2-107-01) and followed the Declaration of Helsinki.

### Calculation of the triglyceride-glucose index

The fasting blood samples were obtained after overnight fasting (> 8 h) and then immediately transferred to the central laboratory of Beijing Friendship Hospital for FPG and TGs measurements following standard laboratory methods. The TyG index was calculated as Ln (fasting TGs [mg/dL] × FPG [mg/dL]/2) [10]. This study applied the TyG index as a continuous variable as well as a category variable based on the TyG index tertiles (T1 < 8.746, T2 = 8.746‒9.336, T3 > 9.336).

### Measurements of NT-proBNP

NT-proBNP levels were measured using the Chemiluminescent Enzyme Immuno Assay (PATHFAST™ Immunoanalyzer, PHC Europe B.V.). The lower and upper limits of detection for this assay are 15 pg/ml and 30,000 pg/ml, respectively, with the coefficient of variation ranging between 4.6% and 5.4%. For further analyses, the peak value of NT-proBNP was used. According to previous studies [25, 26], NT-proBNP was categorized based on the NT-proBNP tertiles (T1 < 129 pg/ml, T2 = 129‒729 pg/ml, T3 > 729 pg/ml).

### Outcomes

Medical record review validated incident cardiovascular events during hospitalization. Follow-up information was collected by telephone interviews or outpatient clinic visits for each participant at 1, 3, and 6 months post-discharge, and annually until the point of their primary endpoint or the end of follow-up (31 March 2021), whichever came first. The primary endpoint of the study was major adverse cardio-cerebral events (MACCEs), which is the composite of all-cause death, non-fatal myocardial infarction, non-fatal stroke, as well as ischemia-induced revascularization. Non-fatal stroke, comprising ischemic and hemorrhagic stroke, was defined as neurological dysfunction caused by cerebral vascular obstruction or sudden rupture, as determined by computed tomography or magnetic resonance imaging. Any revascularization was defined as percutaneous intervention or CABG of the target vessels or non-target vessels.

### Covariates

Clinical characteristics on demographic information, lifestyles, medical history, physical examination, and in-hospital therapies were collected from medical records. Medical history, including previous diagnoses of hypertension, dyslipidemia, myocardial infarction, stroke, arrhythmia, and percutaneous coronary intervention (PCI), was obtained through self-reporting by the patient. In-hospital therapies included PCI, antiplatelet therapy (aspirin, or clopidogrel/ticagrelor), β-blocker, angiotensin-converting enzyme inhibitor (ACEI) or angiotensin receptor blocker (ARB), and statins. BMI was calculated as weight in kilograms divided by height in meters squared. Peripheral venous blood samples were obtained and examined for biomarkers including total cholesterol (TC), low-density lipoprotein cholesterol (LDL-C), high-density lipoprotein cholesterol (HDL-C), HbA1c, high-sensitivity C-reactive protein (hs-CRP), hemoglobin, and serum creatinine. The eGFR was calculated using the Modification of Diet in Renal Disease (MDRD) formula: eGFR (mL/min/1.73 m^2^) = 175 × (Scr)^−1.154^ × (Age)^−0.203^ × (0.742 if female) × (1.212 if African American) [27]. Echocardiograms were performed by expert cardiologists or ultrasound specialists with the LVEF measured using the Simpsons method. Coronary angiography and PCI procedures were implemented by experienced cardiologists according to relevant guidelines. The PCI status was classified into three categories: no PCI performed, timely PCI, and other PCI. For patients diagnosed with STEMI, timely primary PCI was defined as PCI within 90 min of first medical assessment [28]. Timely PCI for patients with NSTEMI or UAP was defined as PCI within two hours for patients at very high risk, and within 24 h for patients at high risk [29].

The Global Registry of Acute Coronary Events (GRACE) risk score was employed to assess the short-term and long-term prognosis. The GRACE risk criteria used to assess the short-term prognosis includes age, heart rate, SBP, Killip class, initial serum creatinine, cardiac arrest on admission, elevated cardiac markers, and ST-segment deviation [30]. The GRACE risk criteria used to assess the long-term prognosis includes age, heart rate, SBP, initial serum creatinine, history of congestive heart failure, history of myocardial infarction, elevated cardiac markers, ST-segment depression, and no in-hospital PCI [31].

### Statistical analysis

Baseline characteristics were presented across three categories of TyG index (< 8.746, 8.746‒9.336, > 9.336) and NT-proBNP (< 129, 129‒729, > 729 pg/ml) as mean ± SD or median (interquartile range [IQR]) for continuous variables and n (%) for categorical variables. One-way ANOVA, Kruskal–Wallis H test, or Pearson χ^2^ test was used to compare the characteristics of these groups.

Crude incidence rates of MACCEs per 1000 person-years were estimated as the number of events divided by total follow-up time. To depict detailed descriptions of the dose–response relationship of the TyG index and NT-proBNP with the incidence of MACCEs, we utilized restricted cubic splines based on flexible parametric survival models (stpm2 package for Stata [version 17.0]) [32]. Wald χ^2^ tests were used to evaluate the statistical significance (at the 0.05 level) of the overall association and the nonlinearity of the risk curves. Flexible parametric survival models were chosen because they are more efficient than the Cox proportional hazards model, and they provide incidence estimates as well as hazard ratios (HRs) with 95% confidence intervals (CIs). According to the findings from the restricted cubic splines analyses (Fig. 1), a nonlinear relationship was observed between NT-proBNP and the incidence of MACCEs (*P*_nonlinear_ < 0.001), whereas the relationship between the TyG index and the incidence of MACCEs was linear (*P*_nonlinear_ > 0.05). Thus, in the independent analyses, NT-proBNP was fitted as a categorical variable, while the TyG index was used as a linear effect. Two multivariable-adjusted models were conducted in the main analyses. The base model included the TyG index, NT-proBNP, age, and sex. In fully adjusted models, additional covariates included BMI, diagnosis of AMI, history of hypertension, history of dyslipidemia, history of myocardial infarction, history of arrhythmia, SBP, LVEF, eGFR, hs-CRP (log-transformed), LDL-C, smoking status, PCI (none, timely, and other), and in-hospital medication (antiplatelet therapy, β-blocker, ACEI or ARB, and statins). Restricted cubic splines with three knots were used to model all additional continuous variables in the fully adjusted model.

Standardized cumulative incidence curves for MACCEs were plotted based on the tertiles of the TyG index and NT-proBNP, using the fully adjusted model [33]. Stratified analyses among participants were performed by age (< 65 or ≥ 65 years) and sex. Using the flexible parametric survival models, we also estimated the adjusted incidence rates for the MACCEs at 65 years of age (the mean population age) in two groups (1 year or 5 years of follow-up since baseline) in both men and women as a function of the TyG index and NT-proBNP.

The association of the combined TyG index and NT-proBNP categories (nine categories with the lowest tertile of the TyG index and NT-proBNP as reference) with MACCEs was then examined. Statistical interaction between the TyG index and NT-proBNP was evaluated by fitting an interaction term in the base and fully adjusted model. The Wald χ^2^ test was used to further test significant interaction. To simply visualize the joint association, we created four mutually exclusive groups: low TyG index and low NT-proBNP (TyG index ≤ 9.336 with NT-proBNP ≤ 729 pg/ml), low TyG index and high NT-proBNP (TyG index ≤ 9.336 with NT-proBNP > 729 pg/ml), high TyG index and low NT-proBNP (TyG index > 9.336 with NT-proBNP ≤ 729 pg/ml), and high TyG index and high NT-proBNP (TyG index > 9.336 with NT-proBNP > 729 pg/ml). By adjusting for the previously indicated cardiovascular risk variables, flexible parametric survival models were used to calculate the standardized cumulative incidence for MACCEs in these groups.

To assess the prognostic performance of the combination of the TyG index and NT-proBNP, the time-dependent receiver operating characteristic (tROC) curve analysis was performed by adding the combination to the existing clinical model (the short-term or long-term GRACE risk score). The area under the tROC curve (tAUC) was reported at 30 days, 6 months, 1 year, and 5 years [34]. In addition, the continuous net reclassification improvement (NRI) index and the integrated discrimination improvement (IDI) index were also calculated.

The robustness of the results was examined by several sensitivity analyses. First, to reduce potential reverse causality, the fully adjusted analysis was repeated after excluding participants who developed MACCEs within the first years of follow-up. Second, the participants diagnosed with STEMI were excluded from the analyses. Third, the analyses were also run after excluding participants with potential familial hypertriglyceridemia (TGs ≥ 5.65 mmol/L). Fourth, we used X-tile software (version 3.6.1; Yale University, New Haven, CT, USA) to determine the optimal cutoff values of the TyG index and NT-proBNP, which is based on the largest χ^2^ value in the Mantel-Cox test [35]. The fully adjusted analysis was repeated. Finally, to assess the potential effect of unmeasured residual confounding, E-value was calculated [36].

All analyses were performed using Stata software, version 17.0 (StataCorp LP, College Station, TX, USA), and R software, version 4.1.2 (R Foundation for Statistical Computing). All *P* values were 2-sided, and *P* < 0.05 was considered statistically significant.

## Results

### Baseline characteristics of patients

**Fig. 2:**
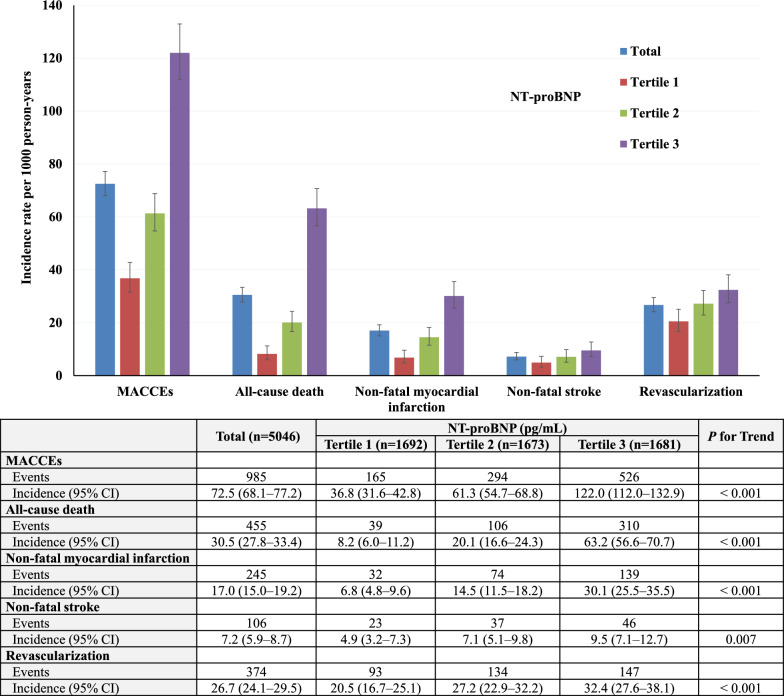
Incidence rates per 1000 person-years of MACCEs, all-cause death, non-fatal myocardial infarction, non-fatal stroke, and revascularization in the whole cohort and stratified by NT-proBNP tertiles

Of the 5046 patients in the current study, the mean age was 65.6 ± 10.6 years, and 62.0% were male. Table 1 shows the baseline characteristics of the study participants by tertiles of NT-proBNP. Figure 2 illustrates the absolute number and incidence per 1000 person-years of composite MACCEs as well as individual events by NT-proBNP groups. During 13589.9 person-years of follow-up, 985 (19.5%) patients experienced a first MACCEs (72.5 per 1000 person-years). The number of both composite MACCEs and individual events increased with elevated NT-proBNP (*P*_trend_ < 0.05). Baseline characteristics by tertiles of the TyG index are shown in Additional file 1: Table S1.

**Table 1:** Baseline and clinical characteristics by NT-proBNP categories

	Total (n = 5046)	NT-proBNP			*P* value
		Tertile 1 (n = 1692)	Tertile 2 (n = 1673)	Tertile 3 (n = 1681)	
Peak value of NT-proBNP, pg/mL	279.0 (90.0, 1331.0)	58.6 (35.1, 90.5)	280.0 (189.0, 438.0)	2421.0 (1331.0, 5596.0)	< 0.001
Clinical characteristics					
Age, year	65.6 ± 10.6	62.1 ± 9.0	66.4 ± 10.4	68.2 ± 11.5	< 0.001
Male, n (%)	3127 (62.0)	1146 (67.7)	987 (59.0)	994 (59.1)	< 0.001
BMI, kg/m^2^	26.1 ± 3.5	26.5 ± 3.4	26.3 ± 3.5	25.5 ± 3.6	< 0.001
Heart rate, bpm	73.6 ± 13.4	72.4 ± 10.8	71.0 ± 11.5	77.5 ± 16.3	< 0.001
SBP, mm Hg	132.9 ± 19.0	131.1 ± 15.9	135.4 ± 19.0	132.3 ± 21.5	< 0.001
Previous hypertension, n (%)	3783 (75.0)	1234 (72.9)	1322 (79.0)	1227 (73.0)	< 0.001
Previous dyslipidemia, n (%)	2494 (49.4)	901 (53.3)	850 (50.8)	743 (44.2)	< 0.001
Previous MI, n (%)	469 (9.3)	100 (5.9)	153 (9.1)	216 (12.8)	< 0.001
Previous arrhythmia	499 (9.9)	101 (6.0)	169 (10.1)	229 (13.6)	< 0.001
Current smoker, n (%)	1668 (33.1)	586 (34.6)	521 (31.1)	561 (33.4)	0.009
LVEF, %	62.8 ± 9.1	67.1 ± 5.4	64.9 ± 7.1	56.6 ± 10.3	< 0.001
ACS status, n (%)					< 0.001
UA	3247 (64.3)	1574 (93.0)	1236 (73.9)	437 (26.0)	
NSTEMI	930 (18.4)	88 (5.2)	281 (16.8)	561 (33.4)	
STEMI	869 (17.2)	30 (1.8)	156 (9.3)	683 (40.6)	
Laboratory examinations					
Triglyceride-glucose index	9.09 ± 0.70	9.08 ± 0.72	9.09 ± 0.69	9.10 ± 0.69	0.72
FPG, mmol/L	7.7 ± 2.8	7.2 ± 2.4	7.5 ± 2.6	8.3 ± 3.2	< 0.001
HbA1c, %	7.5 ± 1.5	7.5 ± 1.4	7.5 ± 1.5	7.6 ± 1.6	0.035
eGFR, ml/min/1.73m^2^	111.7 ± 33.5	123.0 ± 30.2	111.7 ± 31.4	100.6 ± 35.1	< 0.001
Hs-CRP, mg/L	2.4 (0.9, 8.8)	1.3 (0.6, 3.2)	2.0 (0.8, 5.7)	7.5 (2.2, 20.4)	< 0.001
Total cholesterol, mmol/L	4.2 ± 1.1	4.1 ± 1.1	4.2 ± 1.1	4.4 ± 1.1	< 0.001
LDL-C, mmol/L	2.4 ± 0.8	2.3 ± 0.7	2.4 ± 0.8	2.5 ± 0.8	< 0.001
HDL-C, mmol/L	1.0 ± 0.3	1.1 ± 0.2	1.1 ± 0.3	1.0 ± 0.3	0.36
Triglycerides, mmol/L	1.4 (1.1, 2.1)	1.5 (1.1, 2.2)	1.5 (1.1, 2.1)	1.4 (1.0, 2.0)	< 0.001
In-hospital treatment, n (%)					
Aspirin	4558 (90.3)	1541 (91.1)	1536 (91.8)	1481 (88.1)	< 0.001
Clopidogrel/Ticagrelor	3235 (64.1)	864 (51.1)	1070 (64.0)	1301 (77.4)	< 0.001
β-Blocker	3554 (70.4)	1111 (65.7)	1167 (69.8)	1276 (75.9)	< 0.001
ACEI/ARB	2960 (58.7)	853 (50.4)	1030 (61.6)	1077 (64.1)	< 0.001
Statins	4453 (88.2)	1520 (89.8)	1477 (88.3)	1456 (86.6)	0.015
PCI status, n (%)					< 0.001
No PCI performed	2100 (41.6)	875 (51.7)	677 (40.5)	548 (32.6)	
Timely PCI	557 (11.0)	31 (1.8)	121 (7.2)	405 (24.1)	
Other PCI	2389 (47.3)	786 (46.5)	875 (52.3)	728 (43.3)	

Values are mean ± SD, n (%), or median (interquartile interval)

NT-proBNP, N-terminal pro-B-type natriuretic peptide; BMI, body mass index; SBP, systolic blood pressure; MI, myocardial infarction; LVEF, left ventricular ejection fraction; ACS, acute coronary syndrome; UA, unstable angina; NSTEMI, non-ST-segment elevation myocardial infarction; STEMI, ST-segment elevation myocardial infarction; NT-proBNP, N-terminal pro-B-type natriuretic peptide; FPG, fasting plasma glucose; HbA1c, glycosylated hemoglobin; eGFR, estimated glomerular filtration rate; hs-CRP, high sensitivity C-reactive protein; LDL-C, low-density lipoprotein cholesterol; HDL-C, high-density lipoprotein cholesterol; ACEI, angiotensin-converting enzyme inhibitor; ARB, angiotensin receptor blocker; PCI, percutaneous coronary intervention

### Independent associations of the TyG index and NT-proBNP with MACCEs

Table 2 shows the independent associations of the TyG index and NT-proBNP with the risk of MACCEs. In the base model, which included age, sex, the TyG index, and NT-proBNP simultaneously, it was found that both the categorical NT-proBNP and the continuous TyG index were independently associated with an increased risk of MACCEs. These independent associations remained statistically significant in the fully adjusted model. The HRs of the TyG index and NT-proBNP for each individual event are shown in Additional file 1: Table S2. In categorical analyses, the TyG index tertile 3 (HR: 1.28; 95% CI: 1.06‒1.54) and NT-proBNP tertile 3 (HR: 1.94; 95% CI: 1.49‒2.53) had an independent association with MACCEs risk in the fully adjusted model. Additional file 1: Figure S2 shows the corresponding standardized cumulative incidence curves for MACCEs by the TyG index and NT-proBNP categories in the fully adjusted model.

**Table 2:** Independent association of triglyceride-glucose index and NT-proBNP categories with incident MACCEs

	Base Model†		Adjusted Model††	
	Hazard Ratio (95% CI)	*P* Value	Hazard Ratio (95% CI)	*P* Value
NT-proBNP		< 0.001		< 0.001
Tertile 1	1.00 (Reference)		1.00 (Reference)	
Tertile 2	1.53 (1.27‒1.86)	< 0.001	1.30 (1.04‒1.63)	0.020
Tertile 3	2.89 (2.41‒3.45)	< 0.001	1.95 (1.50‒2.54)	< 0.001
TyG index, per 1 unit*	1.16 (1.05‒1.27)	0.002	1.18 (1.05‒1.32)	0.004

^*^Modeled as linear effects

^†^Base model included the TyG index, NT-proBNP, age, and sex

^††^Adjusted model included base model plus BMI, diagnosis of AMI, history of hypertension, history of dyslipidemia, history of myocardial infarction, history of arrhythmia, SBP, LVEF, eGFR, hs-CRP, LDL-C, smoking status, and in-hospital treatments (PCI, antiplatelet therapy, β-blocker, ACEI or ARB, and statins)

NT-proBNP, N-terminal pro-B-type natriuretic peptide; MACCEs, major adverse cardio-cerebral events; CI, confidence interval; TyG, triglyceride-glucose; BMI, body mass index; AMI, acute myocardial infarction; SBP, systolic blood pressure; LVEF, left ventricular ejection fraction; eGFR, estimated glomerular filtration rate; hs-CRP, high sensitivity C-reactive protein; LDL-C, low-density lipoprotein cholesterol; PCI, percutaneous coronary intervention; ACEI, angiotensin-converting enzyme inhibitor; ARB, angiotensin receptor blocker

### Joint associations of the TyG index and NT-proBNP with MACCEs

**Fig. 3:**
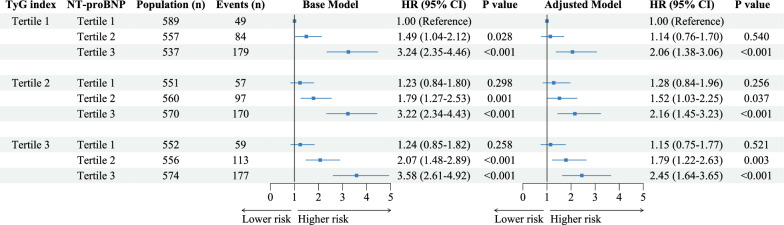
The joint association of the TyG index and NT-proBNP categories with incident MACCEs in the base and fully adjusted model

**Fig. 4:**
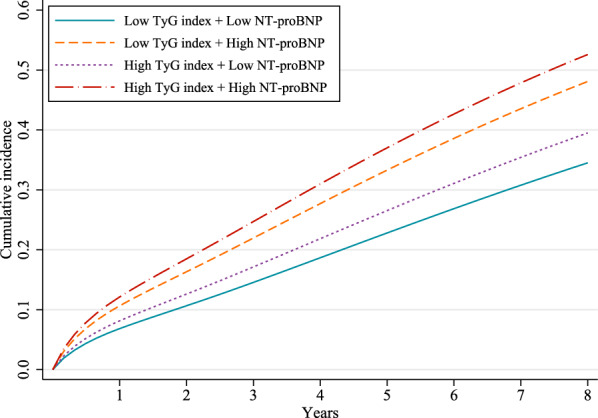
Standardized cumulative incidence curves for MACCEs by the combination of the TyG index and NT-proBNP categories in the fully adjusted model

Figure 3 illustrates the joint associations of the TyG index and NT-proBNP categories with MACCEs risk in the base model and fully adjusted model. Patients in the highest tertile of the TyG index and NT-proBNP had almost a 2.5-fold (HR: 2.45; 95% CI 1.64‒3.65) increased risk of MACCEs compared to those in the lowest tertile of both factors. Multiplicative interaction between the TyG index and NT-proBNP was not significant (*P*_interaction_ = 0.49 in the adjusted model). Figure 4 shows the corresponding standardized cumulative incidence curves for MACCEs by the combination of the TyG index and NT-proBNP categories. Notably, patients with TyG index > 9.336 and NT-proBNP > 729 pg/ml had the highest MACCEs incidence.

### Risk prediction of MACCEs by the TyG index and NT-proBNP

**Fig. 5:**
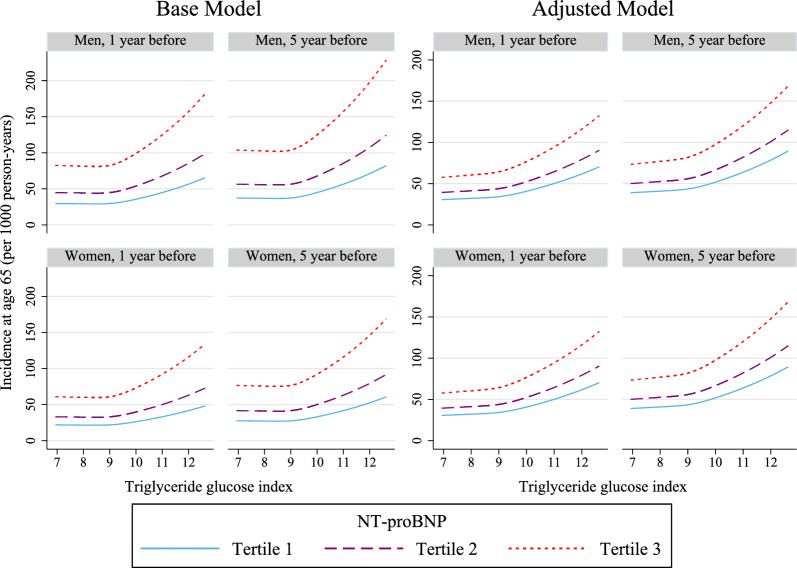
Incidence rate for MACCEs by NT-proBNP categories and the continuous TyG index in the base and fully adjusted model

Figure 5 displays the estimated annual MACCEs incidence rates per 1000 person-years for all men and women at 65 years of age, grouped by follow-up period (1 and 5 years), as well as NT-proBNP categories as a function of the TyG index in the base model (left) and fully adjusted model (right). A higher TyG index was associated with higher annual MACCEs incidence rates in men and women in all two follow-up periods and in all three NT-proBNP groups. Higher NT-proBNP was associated with a higher annual MACCEs incidence rate in both two follow-up periods and continuous levels of the TyG index.

### Predictive value of the TyG index and NT-proBNP compared with GRACE Score

Table 3 shows the discrimination of MACCEs risk for the combinations of the TyG index and NT-proBNP with the GRACE score. Adding these two biomarkers into the model significantly improved risk stratification beyond the GRACE score by improvements in the tAUC, NRI, and IDI (all *P* < 0.05).

**Table 3:** Improvement in MACCEs risk prediction by adding the triglyceride-glucose index and NT-proBNP to the clinical model of GRACE score

	tAUC (95% CI)	*P* Value	NRI (95% CI)	*P* Value	IDI (95% CI)	*P* Value
30 Days MACCEs						
Short-term GRACE score	0.66 (0.60‒0.73)	Reference	Reference		Reference	
Short-term GRACE score + TyG index + NT-proBNP	0.73 (0.68‒0.79)	0.011	0.205 (0.048‒0.305)	< 0.001	0.0026 (0.0001‒0.0059)	0.020
6 Months MACCEs						
Long-term GRACE score	0.62 (0.59‒0.66)	Reference	Reference		Reference	
Long-term GRACE score + TyG index + NT-proBNP	0.70 (0.67‒0.73)	< 0.001	0.271 (0.210‒0.328)	< 0.001	0.015 (0.010‒0.020)	< 0.001
1 Year MACCEs						
Long-term GRACE score	0.60 (0.56‒0.63)	Reference	Reference		Reference	
Long-term GRACE score + TyG index + NT-proBNP	0.67 (0.64‒0.70)	< 0.001	0.233 (0.180‒0.285)	< 0.001	0.019 (0.013‒0.025)	< 0.001
5 Years MACCEs						
Long-term GRACE score	0.61 (0.59‒0.64)		Reference		Reference	
Long-term GRACE score + TyG index + NT-proBNP	0.64 (0.61‒0.66)	0.019	0.104 (0.056‒0.147)	< 0.001	0.025 (0.015‒0.036)	< 0.001

MACCEs, major adverse cardio-cerebral events; NT-proBNP, N-terminal pro-B-type natriuretic peptide; GRACE, The Global Registry of Acute Coronary Events; tAUC, time-dependent area under the receiver operating characteristic curve; CI, confidence interval; NRI, net reclassification improvement index; IDI, integrated discrimination improvement index; TyG, triglyceride-glucose index

### Subgroup and sensitivity analyses

Due to the association of the TyG index and NT-proBNP varied by sex and age (Additional file 1: Figures S3 and S4), subgroup analyses were conducted by sex and age (Additional file 1: Tables S3 and S4). The relationship between the TyG index and NT-proBNP categories with MACCEs remains largely unchanged in a series of sensitivity analyses (Additional file 1: Tables S5, S6, and S7). The estimated E-value based on the fully adjusted model is shown in Additional file 1**: **Figure S5 and S6.

## Discussion

In this study, we assess the independent and joint associations of the TyG index and NT-proBNP with MACCEs risk among patients with diabetes and ACS. The results demonstrated that elevated TyG index or NT-proBNP were independently associated with incident MACCEs. The association of these two markers with subsequent MACCEs risk was independent of each other, as well as other potential confounding factors. Moreover, when the TyG index and NT-proBNP were jointly considered, patients with both elevated TyG index and NT-proBNP had a significantly higher MACCEs risk than those who had neither marker elevated.

### Independent associations of the TyG index and NT-proBNP with the adverse outcome

In patients with established diabetes and ACS, previous studies have demonstrated an independent association between the TyG index and incident cardiovascular events. In a study of 776 patients diagnosed with T_2_DM and ACS who underwent PCI, a significant association was found between the TyG index and adverse cardiovascular outcomes after adjusting for potential confounders [37]. Additionally, it has been shown that the TyG index is positively associated with MACCEs in patients with diabetes who underwent coronary angiography for ACS [16]. Furthermore, according to our previous study, a higher TyG index level was a strong independent predictor of MACCEs in diabetes and AMI patients [15].

Similarly, the independent association of NT-proBNP with an adverse outcome is well-established. Ciardullo et al. demonstrated that NT-proBNP is independently associated with all-cause and cardiovascular mortality in the general population and could help identify individuals at the highest risk of adverse outcomes [38]. In addition, it has been shown that NT-proBNP, soluble suppression of tumorigenesis 2 (sST2), and hs-cTnI are associated with 15-year mortality and the onset of cardiovascular events in T_2_DM [39]. Gerstein et al. also analyzed several validated protein biomarkers and discovered that NT-proBNP independently predicted cardiovascular outcomes, with the addition of other biomarkers only marginally increasing its prognostic value [40]. Moreover, NT-proBNP can be pragmatically used as a screening tool for heart failure and cardiovascular disease risk in T_2_DM and hypertension [41]. Therefore, we consider that the associations of elevated TyG index level and NT-proBNP with MACCEs risk are independent of traditional cardiovascular risk factors.

Besides, one important discovery in this study is that the association of these two markers with subsequent MACCEs risk was independent of each other in the main analysis. This is inconsistent with a previous study which indicated that IR, assessed by HOMA-IR, was not significantly associated with the risk of death or MACCEs in T_2_DM and ACS patients after additional adjustment for NT-proBNP [42]. However, we have found sex differences in the relationship of the TyG index with MACCEs. Further cohort studies are needed to explore the relationship between IR and NT-proBNP and their impact on prognosis.

### Joint associations of the TyG index and NT-proBNP with the adverse outcome

Another novel discovery of the study is that there was an additive joint association between these two markers, where patients with higher levels of the TyG index and NT-porBNP were at an increased risk for incident MACCEs than either risk factor alone. In the current study, it was observed that when the TyG index was ≥ 8.746, the risk of MACCEs was significantly higher in patients with NT-proBNP levels ≥ 129 pg/ml. This finding demonstrates that patients with elevated TyG index levels may have a higher risk of MACCEs if they exhibit mildly elevated NT-proBNP levels, which helps identify patients at a much higher risk. In addition, the combination of these two biomarkers with the GRACE score improves risk stratification. These findings may provide significant prognostic information that patients with both higher TyG index and NT-proBNP levels should be aware of the increased risk of developing MACCEs in the future and may benefit more from proactive MACCEs risk reduction measures.

### Potential mechanisms

The underlying mechanisms of these findings remain debatable. The IR and NT-proBNP levels have a strong inverse relationship that is well recognized [19, 20, 42, 43], which may lead to confusion regarding their respective prognostic significance. In the current study, after adjusting for NT-proBNP and other potential confounders, the subgroup analysis showed that the association between the TyG index and MACCEs was no longer apparent in the male subgroup. Correspondingly, a correlation between lower NT-proBNP and increased TyG index was also observed only in male patients. The paradoxical relationship is probably explained by the interaction between adipose tissue and natriuretic peptides that would accelerate lipolysis, resulting in body weight loss and subsequently improvements in IR [24, 44]. Similarly, recent epidemiological studies have reported a paradoxical association between high circulating levels of adiponectin and increased risk of cardiovascular and all-cause mortality [45, 46]. However, Mukama et al. found that there was a statistically significant effect modification by NT-proBNP for the association between adiponectin and all-cause mortality such that higher hazards of mortality were observed in the context of high NT-proBNP, probably explaining the adiponectin paradox [47]. Therefore, generally, it can be assumed that the prognostic effect of the IR may be influenced by its interaction with NT-proBNP, and there may be a sex difference in this analysis.

On the other hand, previous studies have suggested that the TyG index might reflect cardiac remodeling and dysfunction [48–50]. Chiu et al. assessed the associations between the TyG index and echocardiographic parameters and observed that a high TyG index was associated with a decline in LVEF [48]. It has also been proposed that IR and excess fatty acid contribute to the deposition of intramyocardial lipids, leading to myocardial fibrosis and systolic dysfunction [49]. Moreover, IR may cause the accumulation of intracellular toxic metabolites which can trigger several pathologic signaling pathways, inflammation, production of reactive oxygen species, and mitochondrial dysfunction, thereby inducing cardiac hypertrophy and diastolic dysfunction [50]. Therefore, increased IR may be associated with several other factors which cause the deterioration of the disease, reflected by the elevation of NT-proBNP even though with a potential role in lowering the plasma NT-proBNP levels. However, in each case, the distinction between cause and effect is still difficult. The relationships between IR, NT-proBNP, and cardiovascular outcomes are complex and require further investigation.

### Strengths and limitations

The strengths of the study include its prospective design, large sample size, long follow-up period, as well as the availability of a wide variety of covariates. This could be the first study to explore the independent and joint association of the TyG index and NT-proBNP with subsequent risk of MACCEs in a Chinese cohort of diabetes and ACS patients. However, several limitations should be acknowledged. First, the medical record did not indicate a subtype of diabetes. However, a previous study revealed that T_2_DM in adults accounts for over 90% of diabetes cases [51]. Therefore, the potential existence of the type 1 diabetes population might have limited influence. Second, because there was only one NT-proBNP measurement in the analyses, potential bias from measurement error should be considered. Third, conventional laboratory testing methods for IR, such as HOMA-IR or HIEC, were not routinely collected in the current study. Thus, the relationship between the TyG index and IR could not be evaluated in the present study. Fourth, the utilization of sodium-glucose cotransporter 2 inhibitors (SGLT2i) and glucagon-like peptide 1 receptor agonists (GLP-1 RA) were not included due to their low proportion in our study. Fifth, we did not routinely collect urine albumin-creatinine ratio (UACR) in the present study. Instead, we used the eGFR and the hs-CRP to adjust renal function and inflammation. Finally, given the observational nature of the present study, residual or unmeasured confounders could not be entirely excluded. Further prospective cohort and mechanistic studies are needed to validate our findings.

## Conclusions

In conclusion, our results indicated that the TyG index and NT-proBNP were independently associated with MACCEs risk among patients with diabetes and ACS. The additive joint association between these two markers suggested that patients who had both higher levels of the TyG index and NT-porBNP were at a significantly higher risk for incident MACCEs.

## Supplementary Information

**Additional file 1: Figure S1.** Flowchart of the participants selection. **Figure S2.** Standardized cumulative incidence curves for MACCEs by the TyG indexand NT-proBNPcategories in the fully adjusted model. **Figure S3.** Association between TyG index and NT-proBNP in men and women. **Figure S4.** Association between TyG index and NT-proBNP stratified by age subgroups. **Figure S5.** E-value for MACCEs according to the TyG index for the adjusted model. **Figure S6.** E-value for MACCEs according to NT-proBNP for the adjusted model. **Table S1.** Baseline and clinical characteristics by triglyceride-glucose index categories. **Table S2.** Estimated hazard ratios for all-cause mortality, non-fatal myocardial infarction, non-fatal stroke, and revascularization. **Table S3.** Estimated hazard ratios for MACCEs stratified by sex. **Table S4.** Estimated hazard ratios for MACCEs stratified by age subgroups. **Table S5.** Sensitivity analyses for the independent association of triglyceride-glucose index and NT-proBNP categories with incident MACCEs. **Table S6. **Sensitivity analysis 4 for the independent association of triglyceride-glucose index and NT-proBNP categories divided by the optimal cutoff value with incident MACCEs. **Table S7.** Sensitivity analyses for the joint association of triglyceride-glucose index and NT-proBNP categories with MACCEs risk in the fully adjusted model.

## Data Availability

The datasets used for the present analysis may be made available upon reasonable request by contacting the corresponding author.
