# Supplementary material for: Independent and joint associations between the triglyceride-glucose index and NT-proBNP with the risk of adverse cardiovascular events in patients with diabetes and acute coronary syndrome: a prospective cohort study
Source: Cardiovasc Diabetol. 2023 Jun 26;22:149. doi: 10.1186/s12933-023-01890-9 (PMC10294423; doi:10.1186/s12933-023-01890-9)
Supplement: Supplementary file 1 — Additional file 1: Figure S1. Flowchart of the participants selection. Figure S2. Standardized cumulative incidence curves for MACCEs by the TyG indexand NT-proBNPcategories in the fully adjusted model. Figure S3. Association between TyG index and NT-proBNP in men and women. Figure S4. Association between TyG index and NT-proBNP stratified by age subgroups. Figure S5. E-value for MACCEs according to the TyG index for the adjusted model. Figure S6. E-value for MACCEs according to NT-proBNP for the adjusted model. Table S1. Baseline and clinical characteristics by triglyceride-glucose index categories. Table S2. Estimated hazard ratios for all-cause mortality, non-fatal myocardial infarction, non-fatal stroke, and revascularization. Table S3. Estimated hazard ratios for MACCEs stratified by sex. Table S4. Estimated hazard ratios for MACCEs stratified by age subgroups. Table S5. Sensitivity analyses for the independent association of triglyceride-glucose index and NT-proBNP categories with incident MACCEs. Table S6. Sensitivity analysis 4 for the independent association of triglyceride-glucose index and NT-proBNP categories divided by the optimal cutoff value with incident MACCEs. Table S7. Sensitivity analyses for the joint association of triglyceride-glucose index and NT-proBNP categories with MACCEs risk in the fully adjusted model. [file 12933_2023_1890_MOESM1_ESM.docx]

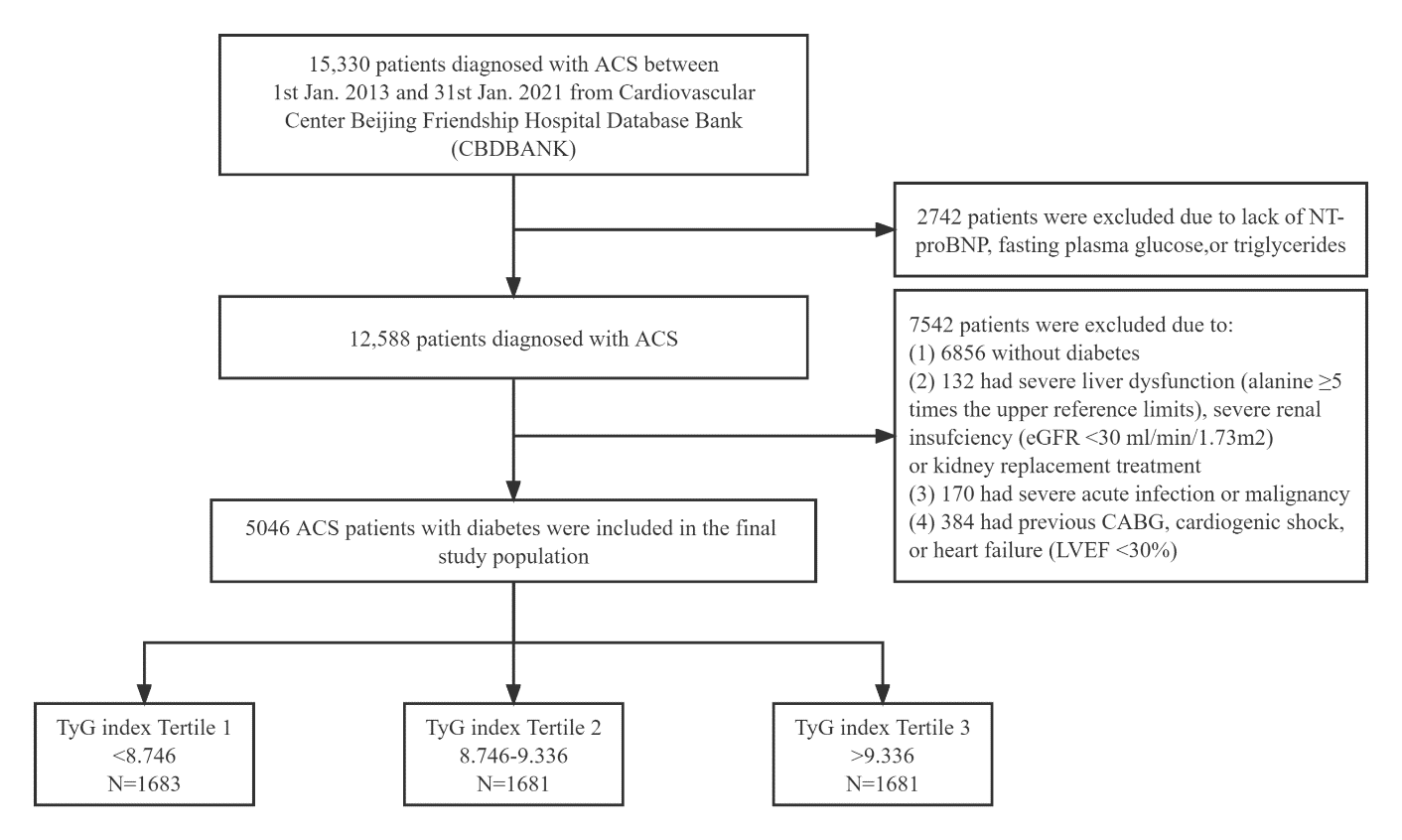


**Supplemental Figure 1.** Flowchart of the participants selection


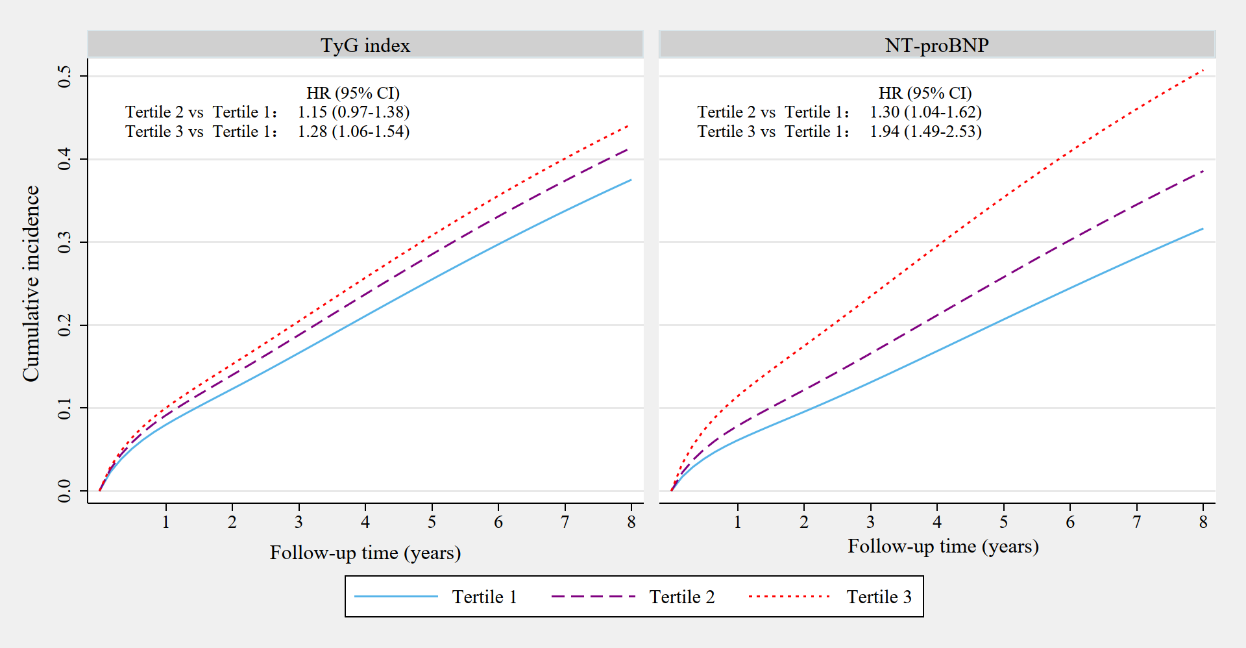


**Additional Figure 2.** Standardized cumulative incidence curves for MACCEs by the TyG index (left) and NT-proBNP (right) categories in the fully adjusted model (including the TyG index, NT-proBNP, age, sex, BMI, diagnosis of AMI, history of hypertension, history of dyslipidemia, history of myocardial infarction, history of arrhythmia, SBP, LVEF, eGFR, hs-CRP, LDL-C, smoking status, and in-hospital treatments (PCI, antiplatelet therapy, β-blocker, ACEI or ARB, and statins).

**Additional Figure 3**. Association between TyG index and NT-proBNP in men and women. Abbreviations: NT-proBNP, N-terminal pro-B-type natriuretic peptide; TyG, triglyceride-glucose.

**Additional Figure 4**. Association between TyG index and NT-proBNP stratified by age subgroups. Abbreviations: NT-proBNP, N-terminal pro-B-type natriuretic peptide; TyG, triglyceride-glucose.

**Additional Figure 5**. E-value for MACCEs according to the TyG index for the adjusted model. Abbreviations: TyG, triglyceride-glucose; MACCEs, major adverse cardio-cerebral events.

**Additional Figure 6**. E-value for MACCEs according to NT-proBNP for the adjusted model. Abbreviations: NT-proBNP, N-terminal pro-B-type natriuretic peptide; MACCEs, major adverse cardio-cerebral events.

| **Additional Table 1. Baseline and clinical characteristics by triglyceride-glucose index categories** | | | | | |
| --- | --- | --- | --- | --- | --- |
|  | **Total (n=5046)** | **Triglyceride-glucose index** | | | ***P* value** |
|  |  | **Tertile 1 (n=1683)** | **Tertile 2 (n=1681)** | **Tertile 3 (n=1682)** |  |
| Triglyceride-glucose index | 9.1 ± 0.7 | 8.4 ± 0.3 | 9.0 ± 0.2 | 9.9 ± 0.5 | < 0.001 |
| **Clinical characteristics** |  |  |  |  |  |
| Age, year | 65.6 ± 10.6 | 68.3 ± 9.7 | 65.9 ± 10.3 | 62.4 ± 11.1 | < 0.001 |
| Male, n (%) | 3127 (62.0) | 1035 (61.5) | 1029 (61.2) | 1063 (63.2) | 0.44 |
| BMI, kg/m^2^ | 26.1 ± 3.5 | 25.4 ± 3.5 | 26.2 ± 3.5 | 26.6 ± 3.5 | < 0.001 |
| Heart rate, bpm | 73.6 ± 13.4 | 71.9 ± 13.1 | 73.6 ± 13.2 | 75.4 ± 13.5 | < 0.001 |
| SBP, mm Hg | 132.9 ± 19.0 | 132.3 ± 18.1 | 132.0 ± 18.7 | 134.6 ± 20.1 | < 0.001 |
| Previous hypertension, n (%) | 3783 (75.0) | 1309 (77.8) | 1253 (74.5) | 1221 (72.6) | 0.002 |
| Previous dyslipidemia, n (%) | 2494 (49.4) | 802 (47.7) | 826 (49.1) | 866 (51.5) | 0.081 |
| Previous MI, n (%) | 469 (9.3) | 170 (10.1) | 154 (9.2) | 145 (8.6) | 0.33 |
| Previous arrhythmia | 499 (9.9) | 209 (12.4) | 165 (9.8) | 125 (7.4) | <0.001 |
| Current smoker, n (%) | 1668 (33.1) | 445 (26.4) | 544 (32.4) | 679 (40.4) | < 0.001 |
| LVEF, % | 62.8 ± 9.1 | 63.4 ± 9.2 | 62.8 ± 9.2 | 62.3 ± 8.8 | 0.002 |
| **ACS status, n (%)** |  |  |  |  | < 0.001 |
| UA | 3247 (64.3) | 1230 (73.1) | 1075 (64.0) | 942 (56.0) |  |
| NSTEMI | 930 (18.4) | 267 (15.9) | 320 (19.0) | 343 (20.4) |  |
| STEMI | 869 (17.2) | 186 (11.1) | 286 (17.0) | 397 (23.6) |  |
| **Laboratory examinations** |  |  |  |  |  |
| Peak value of NT-proBNP, pg/mL | 279.0 (90.0, 1331.0) | 252.0 (88.4, 1335.0) | 283.0 (90.5, 1402.0) | 304.5 (92.6, 1272.0) | 0.50 |
| FPG, mmol/L | 7.7 ± 2.8 | 5.9 ± 1.4 | 7.4 ± 1.9 | 9.8 ± 3.1 | < 0.001 |
| HbA1c, % | 7.5 ± 1.5 | 6.9 ± 1.1 | 7.3 ± 1.3 | 8.3 ± 1.7 | < 0.001 |
| eGFR, ml/min/1.73m^2^ | 111.7 ± 33.5 | 110.4 ± 31.9 | 111.6 ± 33.5 | 113.2 ± 35.2 | 0.065 |
| Hs-CRP, mg/L | 2.4 (0.9, 8.8) | 1.6 (0.6, 6.6) | 2.5 (0.9, 9.2) | 3.1 (1.3, 10.2) | < 0.001 |
| Total cholesterol, mmol/L | 4.2 ± 1.1 | 3.8 ± 0.9 | 4.2 ± 1.0 | 4.8 ± 1.2 | < 0.001 |
| LDL-C, mmol/L | 2.4 ± 0.8 | 2.1 ± 0.6 | 2.4 ± 0.7 | 2.7 ± 0.8 | < 0.001 |
| HDL-C, mmol/L | 1.0 ± 0.3 | 1.1 ± 0.3 | 1.0 ± 0.2 | 1.0 ± 0.2 | < 0.001 |
| Triglycerides, mmol/L | 1.4 (1.1, 2.1) | 1.0 (0.8, 1.2) | 1.5 (1.3, 1.8) | 2.4 (1.9, 3.3) | < 0.001 |
| **In-hospital treatment, n (%)** |  |  |  |  |  |
| Aspirin | 4558 (90.3) | 1491 (88.6) | 1524 (90.7) | 1543 (91.7) | 0.007 |
| Clopidogrel/Ticagrelor | 3235 (64.1) | 1017 (60.4) | 1060 (63.1) | 1158 (68.8) | < 0.001 |
| β-Blocker | 3554 (70.4) | 1125 (66.8) | 1181 (70.3) | 1248 (74.2) | < 0.001 |
| ACEI/ARB | 2960 (58.7) | 962 (57.2) | 941 (56.0) | 1057 (62.8) | < 0.001 |
| Statins | 4453 (88.2) | 1474 (87.6) | 1487 (88.5) | 1492 (88.7) | 0.57 |
| **PCI status, n (%)** |  |  |  |  | < 0.001 |
| No PCI performed | 2100 (41.6) | 814 (48.4) | 702 (41.8) | 584 (34.7) |  |
| Timely PCI | 557 (11.0) | 100 (5.9) | 187 (11.1) | 270 (16.1) |  |
| Other PCI | 2389 (47.3) | 769 (45.7) | 792 (47.1) | 828 (49.2) |  |
| Values are mean ± SD, n (%), or median (interquartile interval).  Abbreviations: BMI, body mass index; SBP, systolic blood pressure; MI, myocardial infarction; LVEF, left ventricular ejection fraction; ACS, acute coronary syndrome; UA, unstable angina; NSTEMI, non-ST-segment elevation myocardial infarction; STEMI, ST-segment elevation myocardial infarction; NT-proBNP, N-terminal pro-B-type natriuretic peptide; FPG, fasting plasma glucose; HbA1c, glycosylated hemoglobin; eGFR, estimated glomerular filtration rate; hs-CRP, high sensitivity C-reactive protein; LDL-C, low-density lipoprotein cholesterol; HDL-C, high-density lipoprotein cholesterol; ACEI, angiotensin-converting enzyme inhibitor; ARB, angiotensin receptor blocker; PCI, percutaneous coronary intervention. | | | | | |

| **Additional Table 2. Estimated hazard ratios for all-cause mortality, non-fatal myocardial infarction, non-fatal stroke, and revascularization** | | | | |
| --- | --- | --- | --- | --- |
|  | **Base Model†** | | **Adjusted Model††** | |
|  | **Hazard Ratio (95% CI)** | ***P* Value** | **Hazard Ratio (95% CI)** | ***P* Value** |
| **All-cause mortality** |  |  |  |  |
| NT-proBNP |  | < 0.001 |  | < 0.001 |
| Tertile 1 | 1.00 (Reference) |  | 1.00 (Reference) |  |
| Tertile 2 | 1.79 (1.23-2.59) | 0.002 | 1.74 (1.13-2.69) | 0.012 |
| Tertile 3 | 4.81 (3.42-6.77) | < 0.001 | 3.26 (2.04-5.19) | < 0.001 |
| TyG index, per 1 unit* | 1.09 (0.95-1.26) | 0.232 | 1.23 (1.04-1.46) | 0.015 |
| **Non-fatal myocardial infarction** |  |  |  |  |
| NT-proBNP |  | < 0.001 |  | 0.001 |
| Tertile 1 | 1.00 (Reference) |  | 1.00 (Reference) |  |
| Tertile 2 | 2.13 (1.40-3.24) | < 0.001 | 1.45 (0.92-2.28) | 0.108 |
| Tertile 3 | 4.34 (2.94-6.41) | < 0.001 | 2.50 (1.49-4.21) | 0.001 |
| TyG index, per 1 unit* | 1.35 (1.13-1.62) | 0.001 | 1.23 (1.00-1.52) | 0.051 |
| **Non-fatal stroke** |  |  |  |  |
| NT-proBNP |  | 0.101 |  | 0.488 |
| Tertile 1 | 1.00 (Reference) |  | 1.00 (Reference) |  |
| Tertile 2 | 1.34 (0.79-2.28) | 0.273 | 1.46 (0.78-2.76) | 0.241 |
| Tertile 3 | 1.74 (1.04-2.92) | 0.035 | 1.50 (0.66-3.38) | 0.334 |
| TyG index, per 1 unit* | 0.79 (0.59-1.07) | 0.125 | 0.81 (0.56-1.16) | 0.249 |
| **Revascularization** |  |  |  |  |
| NT-proBNP |  | < 0.001 |  | 0.038 |
| Tertile 1 | 1.00 (Reference) |  | 1.00 (Reference) |  |
| Tertile 2 | 1.47 (1.13-1.92) | 0.005 | 1.26 (0.92-1.73) | 0.156 |
| Tertile 3 | 1.78 (1.36-2.31) | < 0.001 | 1.69 (1.13-2.54) | 0.011 |
| TyG index, per 1 unit* | 1.21 (1.05-1.40) | 0.008 | 1.19 (1.00-1.41) | 0.044 |
| *Modeled as linear effects. | | | | |
| †Base model included the TyG index, NT-proBNP, age, and sex. | | | | |
| ††Adjusted model included base model plus BMI, diagnosis of AMI, history of hypertension, history of dyslipidemia, history of myocardial infarction, history of arrhythmia, SBP, LVEF, eGFR, hs-CRP, LDL-C, smoking status, and in-hospital treatments (PCI, antiplatelet therapy, β-blocker, ACEI or ARB, and statins). | | | | |
| Abbreviations: CI, confidence interval; NT-proBNP, N-terminal pro-B-type natriuretic peptide; TyG, triglyceride-glucose; BMI, body mass index; AMI, acute myocardial infarction; SBP, systolic blood pressure; LVEF, left ventricular ejection fraction; eGFR, estimated glomerular filtration rate; hs-CRP, high sensitivity C-reactive protein; LDL-C, low-density lipoprotein cholesterol; PCI, percutaneous coronary intervention; ACEI, angiotensin-converting enzyme inhibitor; ARB, angiotensin receptor blocker. | | | | |

| **Additional Table 3. Estimated hazard ratios for MACCEs stratified by sex** | | | | | |
| --- | --- | --- | --- | --- | --- |
|  |  | **Base Model** | | **Adjusted Model†** | |
|  |  | **Hazard Ratio (95% CI)** | ***P* Value** | **Hazard Ratio (95% CI)** | ***P* Value** |
| Men | NT-proBNP |  | < 0.001 |  | 0.011 |
|  | Tertile 1 | 1.00 (Reference) |  | 1.00 (Reference) |  |
|  | Tertile 2 | 1.46 (1.16-1.83) | 0.001 | 1.20 (0.92-1.57) | 0.179 |
|  | Tertile 3 | 2.48 (2.00-3.06) | < 0.001 | 1.62 (1.17-2.24) | 0.004 |
|  | TyG index, per 1 unit* | 1.08 (0.96-1.21) | 0.193 | 1.08 (0.94-1.24) | 0.284 |
| Women | NT-proBNP |  | < 0.001 |  | < 0.001 |
|  | Tertile 1 | 1.00 (Reference) |  | 1.00 (Reference) |  |
|  | Tertile 2 | 1.86 (1.27-2.72) | 0.001 | 1.71 (1.10-2.66) | 0.017 |
|  | Tertile 3 | 4.02 (2.80-5.76) | < 0.001 | 3.09 (1.89-5.03) | < 0.001 |
|  | TyG index, per 1 unit* | 1.28 (1.10-1.50) | 0.002 | 1.32 (1.09-1.59) | 0.004 |
| *Modeled as linear effects. | | | | | |
| †Base model included the TyG index, NT-proBNP, and age. | | | | | |
| ††Adjusted model included base model plus BMI, diagnosis of AMI, history of hypertension, history of dyslipidemia, history of myocardial infarction, history of arrhythmia, SBP, LVEF, eGFR, hs-CRP, LDL-C, smoking status, and in-hospital treatments (PCI, antiplatelet therapy, β-blocker, ACEI or ARB, and statins). | | | | | |
| Abbreviations: MACCEs, major adverse cardio-cerebral events; CI, confidence interval; NT-proBNP, N-terminal pro-B-type natriuretic peptide; TyG, triglyceride-glucose; BMI, body mass index; AMI, acute myocardial infarction; SBP, systolic blood pressure; LVEF, left ventricular ejection fraction; eGFR, estimated glomerular filtration rate; hs-CRP, high sensitivity C-reactive protein; LDL-C, low-density lipoprotein cholesterol; PCI, percutaneous coronary intervention; ACEI, angiotensin-converting enzyme inhibitor; ARB, angiotensin receptor blocker. | | | | | |

| **Additional Table 4. Estimated hazard ratios for MACCEs stratified by age subgroups** | | | | | |
| --- | --- | --- | --- | --- | --- |
|  |  | **Base Model** | | **Adjusted Model†** | |
|  |  | **Hazard Ratio (95% CI)** | ***P* Value** | **Hazard Ratio (95% CI)** | ***P* Value** |
| < 65 years | NT-proBNP |  | < 0.001 |  | 0.005 |
|  | Tertile 1 | 1.00 (Reference) |  | 1.00 (Reference) |  |
|  | Tertile 2 | 1.59 (1.21-2.09) | 0.001 | 1.33 (0.95-1.85) | 0.095 |
|  | Tertile 3 | 2.74 (2.13-3.53) | < 0.001 | 1.96 (1.30-2.96) | 0.001 |
|  | TyG index, per 1 unit* | 1.10 (0.95-1.27) | 0.203 | 1.08 (0.91-1.29) | 0.358 |
| ≥ 65 years | NT-proBNP |  | < 0.001 |  | 0.001 |
|  | Tertile 1 | 1.00 (Reference) |  | 1.00 (Reference) |  |
|  | Tertile 2 | 1.46 (1.10-1.94) | 0.009 | 1.28 (0.93-1.77) | 0.128 |
|  | Tertile 3 | 2.78 (2.13-3.63) | < 0.001 | 1.87 (1.30-2.69) | 0.001 |
|  | TyG index, per 1 unit* | 1.16 (1.03-1.32) | 0.016 | 1.24 (1.07-1.43) | 0.005 |
| *Modeled as linear effects. | | | | | |
| †Base model included the TyG index, NT-proBNP, age, and sex. | | | | | |
| ††Adjusted model included base model plus BMI, diagnosis of AMI, history of hypertension, history of dyslipidemia, history of myocardial infarction, history of arrhythmia, SBP, LVEF, eGFR, hs-CRP, LDL-C, smoking status, and in-hospital treatments (PCI, antiplatelet therapy, β-blocker, ACEI or ARB, and statins). | | | | | |
| Abbreviations: MACCEs, major adverse cardio-cerebral events; CI, confidence interval; NT-proBNP, N-terminal pro-B-type natriuretic peptide; TyG, triglyceride-glucose; BMI, body mass index; AMI, acute myocardial infarction; SBP, systolic blood pressure; LVEF, left ventricular ejection fraction; eGFR, estimated glomerular filtration rate; hs-CRP, high sensitivity C-reactive protein; LDL-C, low-density lipoprotein cholesterol; PCI, percutaneous coronary intervention; ACEI, angiotensin-converting enzyme inhibitor; ARB, angiotensin receptor blocker. | | | | | |

| **Additional Table 5. Sensitivity analyses for the independent association of triglyceride-glucose index and NT-proBNP categories with incident MACCEs** | | | | | | |
| --- | --- | --- | --- | --- | --- | --- |
|  | **Sensitivity analysis 1†** | | **Sensitivity analysis 2†** | | **Sensitivity analysis 3†** | |
|  | **Hazard Ratio**  **(95% CI)** | ***P* Value** | **Hazard Ratio**  **(95% CI)** | ***P* Value** | **Hazard Ratio**  **(95% CI)** | ***P* Value** |
| NT-proBNP |  | < 0.001 |  | < 0.001 |  | < 0.001 |
| Tertile 1 | 1.00 (Reference) |  | 1.00 (Reference) |  | 1.00 (Reference) |  |
| Tertile 2 | 1.23 (0.93-1.61) | 0.145 | 1.25 (0.99-1.57) | 0.060 | 1.25 (1.00-1.57) | 0.055 |
| Tertile 3 | 1.94 (1.40-2.70) | < 0.001 | 2.16 (1.63-2.86) | < 0.001 | 1.90 (1.46-2.49) | < 0.001 |
| TyG index, per 1 unit* | 1.19 (1.03-1.37) | 0.017 | 1.17 (1.03-1.32) | 0.015 | 1.14 (1.01-1.28) | 0.040 |
| *Modeled as linear effects. | | | | |  |  |
| †Adjusted model included the TyG index, NT-proBNP, age, sex, BMI, diagnosis of AMI, history of hypertension, history of dyslipidemia, history of myocardial infarction, history of arrhythmia, SBP, LVEF, eGFR, hs-CRP, LDL-C, smoking status, and in-hospital treatments (PCI, antiplatelet therapy, β-blocker, ACEI or ARB, and statins). | | | | | | |
| Abbreviations: NT-proBNP, N-terminal pro-B-type natriuretic peptide; MACCEs, major adverse cardio-cerebral events; CI, confidence interval; TyG, triglyceride-glucose; BMI, body mass index; AMI, acute myocardial infarction; SBP, systolic blood pressure; LVEF, left ventricular ejection fraction; eGFR, estimated glomerular filtration rate; hs-CRP, high sensitivity C-reactive protein; LDL-C, low-density lipoprotein cholesterol; PCI, percutaneous coronary intervention; ACEI, angiotensin-converting enzyme inhibitor; ARB, angiotensin receptor blocker. | | | | | | |

| **Additional Table 6. Sensitivity analysis 4 for the independent association of triglyceride-glucose index and NT-proBNP categories divided by the optimal cutoff value with incident MACCEs** | | |
| --- | --- | --- |
|  | **Hazard Ratio (95% CI) †** | ***P* Value** |
| NT-proBNP |  |  |
| < 354 pg/ml | 1.00 (Reference) |  |
| 354‒4240 pg/ml | 1.41 (1.16-1.71) | < 0.001 |
| > 4240 pg/ml | 2.03 (1.52-2.71) | < 0.001 |
| TyG index |  |  |
| < 8.52 | 1.00 (Reference) |  |
| 8.52‒9.97 | 1.15 (0.95-1.39) | 0.144 |
| > 9.97 | 1.45 (1.09-1.92) | 0.010 |
| †Adjusted model included the TyG index, NT-proBNP, age, sex, BMI, diagnosis of AMI, history of hypertension, history of dyslipidemia, history of myocardial infarction, history of arrhythmia, SBP, LVEF, eGFR, hs-CRP, LDL-C, smoking status, and in-hospital treatments (PCI, antiplatelet therapy, β-blocker, ACEI or ARB, and statins). | | |
| Abbreviations: NT-proBNP, N-terminal pro-B-type natriuretic peptide; MACCEs, major adverse cardio-cerebral events; CI, confidence interval; TyG, triglyceride-glucose; BMI, body mass index; AMI, acute myocardial infarction; SBP, systolic blood pressure; LVEF, left ventricular ejection fraction; eGFR, estimated glomerular filtration rate; hs-CRP, high sensitivity C-reactive protein; LDL-C, low-density lipoprotein cholesterol; PCI, percutaneous coronary intervention; ACEI, angiotensin-converting enzyme inhibitor; ARB, angiotensin receptor blocker. | | |

| **Additional Table 7. Sensitivity analyses for the joint association of triglyceride-glucose index and NT-proBNP categories with MACCEs risk in the fully adjusted model** | | | | | | | | | |
| --- | --- | --- | --- | --- | --- | --- | --- | --- | --- |
|  | | **Sensitivity analysis 1†*** | | **Sensitivity analysis 2†*** | | **Sensitivity analysis 3†*** | | **Sensitivity analysis 4†※** | |
| **TyG index group** | **NT-proBNP group** | **HR (95% CI)** | ***P* Value** | **HR (95% CI)** | ***P* Value** | **HR (95% CI)** | ***P* Value** | **HR (95% CI)** | ***P* Value** |
| Group 1 | Group 1 | 1.00 (Reference) |  | 1.00 (Reference) |  | 1.00 (Reference) |  | 1.00 (Reference) |  |
|  | Group 2 | 1.02 (0.62-1.67) | 0.932 | 1.15 (0.76-1.73) | 0.503 | 1.13 (0.75-1.70) | 0.553 | 2.03 (1.36-3.03) | 0.001 |
|  | Group 3 | 2.04 (1.25-3.31) | 0.004 | 2.33 (1.54-3.52) | < 0.001 | 2.03 (1.36-3.02) | < 0.001 | 2.72 (1.67-4.42) | < 0.001 |
| Group 2 | Group 1 | 1.09 (0.64-1.86) | 0.756 | 1.24 (0.81-1.91) | 0.323 | 1.28 (0.84-1.96) | 0.255 | 1.44 (1.02-2.03) | 0.036 |
|  | Group 2 | 1.39 (0.86-2.24) | 0.182 | 1.42 (0.95-2.12) | 0.089 | 1.51 (1.02-2.24) | 0.039 | 2.02 (1.42-2.89) | < 0.001 |
|  | Group 3 | 2.29 (1.40-3.74) | 0.001 | 2.24 (1.47-3.41) | < 0.001 | 2.12 (1.42-3.17) | < 0.001 | 2.99 (1.98-4.52) | < 0.001 |
| Group 3 | Group 1 | 1.30 (0.79-2.15) | 0.307 | 1.18 (0.77-1.82) | 0.450 | 1.15 (0.74-1.79) | 0.526 | 2.30 (1.50-3.52) | < 0.001 |
|  | Group 2 | 1.77 (1.11-2.81) | 0.016 | 1.69 (1.14-2.52) | 0.009 | 1.65 (1.12-2.45) | 0.012 | 2.19 (1.36-3.50) | 0.001 |
|  | Group 3 | 2.31 (1.41-3.79) | 0.001 | 2.82 (1.84-4.32) | < 0.001 | 2.41 (1.61-3.61) | < 0.001 | 2.63 (1.28-5.39) | 0.008 |
| †Adjusted for age, sex, BMI, diagnosis of AMI, history of hypertension, history of dyslipidemia, history of myocardial infarction, history of arrhythmia, SBP, LVEF, eGFR, hs-CRP, LDL-C, smoking status, and in-hospital treatments (PCI, antiplatelet therapy, β-blocker, ACEI or ARB, and statins). | | | | | | | | | |
| * Grouped by tertiles of the TyG index and NT-proBNP. | | | | | | | | | |
| ※ Grouped by the optimal cutoff value of the TyG index (< 8.52, 8.52‒9.97, and > 9.97) and NT-proBNP (< 354 pg/ml, 354‒4240 pg/ml, and > 4240 pg/ml). | | | | | | | | | |
| Abbreviations: NT-proBNP, N-terminal pro-B-type natriuretic peptide; MACCEs, major adverse cardio-cerebral events; TyG, triglyceride-glucose; CI, confidence interval; BMI, body mass index; AMI, acute myocardial infarction; SBP, systolic blood pressure; LVEF, left ventricular ejection fraction; eGFR, estimated glomerular filtration rate; hs-CRP, high sensitivity C-reactive protein; LDL-C, low-density lipoprotein cholesterol; PCI, percutaneous coronary intervention; ACEI, angiotensin-converting enzyme inhibitor; ARB, angiotensin receptor blocker. | | | | | | | | | |
